# RNA-seq transcriptome profiling reveals that *Medicago truncatula* nodules acclimate N_2_ fixation before emerging P deficiency reaches the nodules

**DOI:** 10.1093/jxb/eru341

**Published:** 2014-08-23

**Authors:** Ricardo A. Cabeza, Rebecca Liese, Annika Lingner, Ilsabe von Stieglitz, Janice Neumann, Gabriela Salinas-Riester, Claudia Pommerenke, Klaus Dittert, Joachim Schulze

**Affiliations:** ^1^Department of Crop Science, Section for Plant Nutrition and Crop Physiology, Faculty of Agriculture, University of Goettingen, Carl-Sprengel-Weg 1, 37075 Goettingen, Germany; ^2^Department of Developmental Biochemistry, DNA Microarray and Deep-Sequencing Facility, Faculty of Medicine, University of Goettingen, Justus-von-Liebig-Weg 11, 37077 Goettingen, Germany

**Keywords:** Legumes, *Medicago truncatula*, nitrogen fixation, N2 fixation, nodulation, P stress, RNA-seq, *Sinorhizobium meliloti*.

## Abstract

Legume nodules are plant tissues with an exceptionally high concentration of phosphorus (P), which, when there is scarcity of P, is preferentially maintained there rather than being allocated to other plant organs. The hypothesis of this study was that nodules are affected before the P concentration in the organ declines during whole-plant P depletion. Nitrogen (N_2_) fixation and P concentration in various organs were monitored during a whole-plant P-depletion process in *Medicago truncatula*. Nodule gene expression was profiled through RNA-seq at day 5 of P depletion. Until that point in time P concentration in leaves reached a lower threshold but was maintained in nodules. N_2_-fixation activity per plant diverged from that of fully nourished plants beginning at day 5 of the P-depletion process, primarily because fewer nodules were being formed, while the activity of the existing nodules was maintained for as long as two weeks into P depletion. RNA-seq revealed nodule acclimation on a molecular level with a total of 1140 differentially expressed genes. Numerous genes for P remobilization from organic structures were increasingly expressed. Various genes involved in nodule malate formation were upregulated, while genes involved in fermentation were downregulated. The fact that nodule formation was strongly repressed with the onset of P deficiency is reflected in the differential expression of various genes involved in nodulation. It is concluded that plants follow a strategy to maintain N_2_ fixation and viable leaf tissue as long as possible during whole-plant P depletion to maintain their ability to react to emerging new P sources (e.g. through active P acquisition by roots).

## Summary

During a whole-plant P-depletion process in *Medicago*, formation of new nodules ceases and leaves become P depleted, while existing active nodules maintain high-P levels and display complex molecular acclimation processes.

## Introduction

Phosphorus (P) is a major plant nutrient but it is estimated that inexpensive and easily accessible rock phosphate deposits will be depleted during the 21st century (Runge-Metzger, 1995; Cordell *et al.*, 2009). A large proportion of soil phosphate is organically bound (Richardson and Simpson, 2011) and inorganic phosphate is subject to complex sorption and precipitation processes (Holford, 1997), resulting in a high P-buffering capacity of soils and a strong soil-pH dependency of P availability for plants (Shen *et al.*, 2011). As a consequence, only a small proportion of soil P is found in the soil solution that is directly accessible to plant roots (Jungk and Claassen, 1997). Thus P is frequently the principal limiting factor for plant productivity across a large proportion of the world’s arable land.

P is a constituent of several macromolecules that are of structural importance for plants, and in various ways is required for information, energy, and substance turnover in plants (Raghothama, 1999). Given that P is crucially important for plant growth, plants have evolved strategies for increasing soil P availability and accessibility through intensive root formation and root turnover under low-P conditions (Steingrobe *et al.*, 2001), making use of the benefits of mycorrhizal symbiosis (Bolan, 1991; Marschner and Dell, 1994; Küster *et al*., 2007) and the root exudation of compounds that increase P availability (Shane and Lambers, 2005), either directly or through bacteria-mediated effects (Schilling *et al.*, 1998; Marschner *et al.*, 2011). Comparative transcriptome and proteome studies reveal complex internal reactions and acclimations of plant organs to low-P concentrations (Hernández *et al.*, 2009; Ramirez *et al.*, 2013), including long-distance signalling processes involving miRNA and sugars as signal carriers (Vance, 2010).

P deficiency constitutes a constraint for nitrogen (N_2_) fixation in legumes, especially in soils with a high P-buffering capacity (Graham *et al.*, 2003). Low-P availability results in slow plant growth and a decrease in N_2_ fixation (Israel, 1987). In low-P soils, legumes depending on N_2_ fixation respond positively to P fertilization and show increased N content in shoots and roots (Robson *et al.*, 1981). Rather than being confronted with a situation of low organ P concentration from the early stages of growth, plants will more often encounter slow P depletion when, for example, the exponential increase of shoot dry matter (DM) formation during ontogeny exceeds soil P supply and alternative mechanisms for increasing soil P availability are lacking. P-limited grain legumes in particular can support normal N_2_ fixation for as long as three weeks solely on the basis of seed P reserves (Schulze *et al.*, 2006). Consequently any promising effort to improve N_2_ fixation under P deficiency should target the primary mechanism that affects growth and nitrogenase activity under conditions of emerging P deficiency. It is an unresolved question as to how far into an emerging process of whole-plant P depletion acclimation processes will support high N_2_-fixation rates, and whether the initial reason for a decline in N_2_ fixation is insufficient P in nodules or an earlier shoot-mediated process.

Legume nodules are sites of intensive carbon (C) and energy turnover, and hence are particularly P-rich plant tissue (Israel, 1987; Israel, 1993). P concentration in nodules can be up to three times that in leaves and roots (Sa and Israel, 1991; Schulze *et al.*, 2006). In addition nodules represent a preferential P sink among the various plant organs. Grown under a continuously low P supply, legumes preferentially allocate the scarce P to nodules, with leaves depleting strongly before the nodule P concentration is significantly affected (Sulieman *et al.*, 2010). In turn, when heavily depleted plants are resupplied with a limited amount of P, nodules rapidly reach sufficient P concentrations (Israel, 1993). The pivotal importance of P in nodules formed the basis for the hypothesis of this study that nodules would acclimate to low-P availability at early stages of whole-plant P depletion in order to adjust N_2_ fixation to changing source–sink relationships within the plant. It was further hypothesized that some of these mechanisms of acclimation are shoot-mediated and induced before the P concentration in nodules is diluted. To test this hypothesis, N_2_ fixation per plant (continuous measurements of H_2_ evolution) and the P concentration in various organs (analysis of plants grown in parallel) were monitored concurrently after cessation of P supply in one treatment (P-free nutrient solution). At the point in time when both treatments began to diverge in N_2_ fixation per plant, an RNA-seq-based transcriptome profiling of the nodules was performed to provide a comprehensive picture of the shoot-mediated molecular mechanisms that acclimate nodules to low whole-plant P availability. In addition, given the importance of leaf P for photosynthesis and the high respiratory cost of N_2_ fixation, an artificial feeding of additional sucrose was applied in order to show whether the system is assimilate limited at early stages of P depletion.

## Material and Methods

### Design of the experiments

**Fig. 1.**
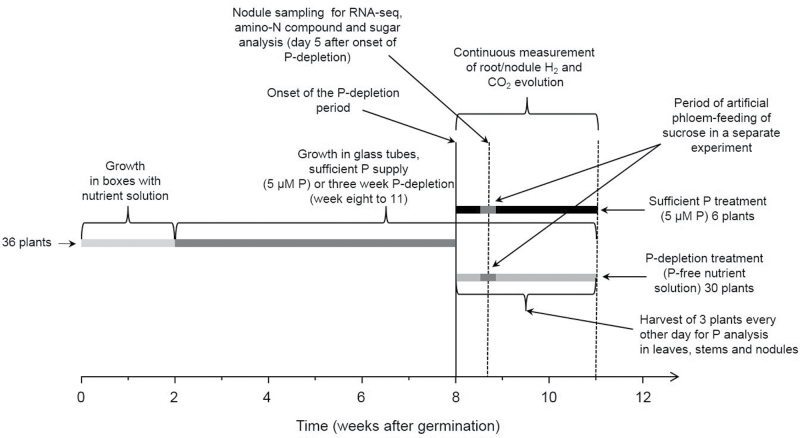
Schematic representation of the experimental design. The periods of time of growth under different conditions are represented by thick lines of a different shades of grey. A total of 36 plants were grown and divided into control (six plants) and P-depletion treatment at the beginning of week 8 of the experiment. A total of three experiments were performed: (1) continuous measurements of nodule activity during P depletion and consecutive harvests for measuring P concentration; (2) artificial phloem feeding; (3) nodule harvest for RNA-seq, amino-N compound, and sugar analysis.

A system for non-invasive measurements of nitrogenase activity (H_2_ evolution) was adapted to run over longer periods of time (weeks) with continuous measurements. The set-up for measuring nodule H_2_ evolution was embedded in a hydroponic growth system with a virtual flow through supply of nutrient solution. This set-up allowed us to impose the P-depletion period through application of P-free nutrient solution without interrupting or disturbing ongoing H_2_-evolution measurements. Hence P dilution in various plant organs (through frequent harvests of plants grown under exactly the same conditions) and N_2_ fixation could be monitored concurrently. The experimental design is shown schematically in Fig. 1. The model legume *Medicago truncatula* was used for these experiments. The completed genome project (Young *et al.*, 2011) and the gene expression atlas (Benedito *et al.*, 2008) provide the ideal prerequisites for analysis of the RNA-seq data. To date, RNA-seq is the most powerful tool for comparative transcriptome profiling and has been used successfully with *M. truncatula* tissues, including nodules (Boscari *et al.*, 2013; Cabeza *et al.*, 2014).

### Plant growth

Seeds of *M. truncatula* (Gaertn.) cv. Jemalong A17 were submerged in H_2_SO_4_ (96%) for 5min for chemical scarification, sterilized with 5% (v/v) sodium hypochlorite for 5min and rinsed several times with deionized water. The seeds were subsequently kept at 4°C for 12h in darkness, submerged in tap water. The next step was to shake the submerged seeds slightly at 25°C and in continuous light for 2–4 days. When the seed had developed a primary root about 20mm long, 20 plantlets were transferred to small growth boxes (170mm × 125mm × 50mm) filled with aerated nutrient solution. The seedlings were fixed through small x-shaped cuts in tape onto the top of the growth boxes. The plants were grown for two weeks in these boxes in a growth chamber with a 16/8h light/dark cycle at 25/20°C, respectively. The nutrient solution level in the boxes was maintained by adding an appropriate amount of nutrient solution every other day. Light intensity at plant height was approximately 500 µmol m^−2^ s^−1^. Immediately after being transferred to the growth boxes, the seedlings were inoculated with 1ml box^–1^ of a stationary *Sinorhizobium meliloti* (Sm) (strain 102F51) YEM-culture, with an approximate cell density of 10^9^ ml^−1^. The Sm-strain induced good nodulation, with the first nodules visible to the naked eye appearing after about 7–10 days. The strain does not contain an H_2_-uptake hydrogenase (Blumenthal *et al*., 1997).

After two weeks, the plants were transferred to glass tubes, allowing the separate measurement of root/nodule H_2_ and CO_2_ evolution. The system is described in Fischinger and Schulze (2010*a*). The set-up was extended by connecting a group of six plants through the lower side of the glass tubes to a 20 l nutrient-solution container. Due to gravity, the nutrient-solution level in the glass tubes depended on the height of the container position, and losses through plant transpiration could be adjusted by the addition of nutrient solution to the container, thereby not interfering with the measurements in the root/nodule compartment. In addition, a pump in the container drove a nutrient solution flow of about 10ml min^–1^ into the upper part of each individual glass tube. Each individual glass tube contained about 150ml nutrient solution, which was turned over every 15min. The nutrient solution in each glass tube was individually aerated with a gas stream of 200ml min^–1^ [N_2_:O_2_ ratio of 80:20 (v/v)]. The nutrient solution contained the macronutrients (mM): K_2_SO_4_, 0.7; MgSO_4_, 0.5; and CaCl_2_, 0.8. It contained the micronutrients (μM): H_3_BO_3_, 4.0; Na_2_MoO_4_, 0.1; ZnSO_4_, 1.0; MnCl_2_, 2.0; CoCl_2_, 0.2; CuCl_2_, 1.0; and FeNaEDTA (ferric monosodium salt of ethylenediamine tetraacetic acid), 30. The pH was adjusted to 6.4 through KOH and buffered with 0.25mM 2-(N-morpholino) ethane-sulfonic acid (MES). P was added daily as KH_2_PO_4_ to a concentration of 5 µM P. Furthermore, in the first week after being transferred to the glass tubes, the nutrient solution was adjusted once to a 0.5mM NH_4_^+^ concentration through the addition of NH_4_SO_4_. Low concentrations of ammonium support nodule formation in *M. truncatula* (Fei and Vessey, 2009). The nutrient solution was changed every week. During this procedure, the pump in the container was switched off and the backflow from the glass tubes to the container was blocked. This meant that ongoing measurements in the root/nodule compartment were not affected. The procedure of replacing the nutrient solution in the container took about ten minutes, after which the nutrient solution turnover system was returned to normal functioning. After the first week of growth in the glass tubes, the plants depended solely on N_2_ fixation for N nutrition.

### Root/nodule gas exchange measurement

The system for measuring nodule H_2_ and CO_2_ evolution, including the determination of apparent nitrogenase activity (ANA), total nitrogenase activity (TNA), the calculation of the electron allocation coefficient (EAC) and the calculation of N_2_ fixation is described in Fischinger and Schulze (2010*a*). For a continuous, long-term measurement of H_2_ evolution the set-up was extended by an efficient three-step air-drying system for the airstream flowing out of the root/nodule compartment.

### P-depletion experiments

Three P-depletion experiments were conducted. After six weeks of growth in the glass tubes (eight weeks after germination) (see Cabeza *et al.*, 2014) the daily adjustment of the nutrient solution to 5 µM PO_4_-concentration for six glass tubes was interrupted (Fig. 1), while another set of six plants was continuously supplied with the full nutrient solution as a control. In a first experiment, a significant difference in ANA between the treatments occurred at day 5 during P depletion. TNA was measured at day 6 and at the end of the experimental period. In a second similar experiment, a subset of three plants in each treatment was supplied with additional sucrose through artificial phloem feeding. In a third experiment, nodules were harvested at day 5 of the P-depletion process for comparative RNA-seq-based transcriptome profiling and determination of nodule sucrose and amino-N compounds.

### Sucrose feeding into the phloem

From days 4–6 of the second experiment, three plants in both treatments were artificially fed with sucrose into the phloem. The method for feeding a solution into the phloem is described by Lin *et al.* (2010) and was adapted for use on *M. truncatula* by Sulieman *et al.* (2010). On average the plants took up 2ml day^-1^ of a 6% (w/v) sucrose solution into the phloem. The cut of the branch stub for the tubing connection to the sucrose solution reservoir was renewed on day 3 of the feeding period after the uptake rates of the solution had fallen.

### RNA extraction, cDNA library preparation, and RNA-seq

RNA extraction, cDNA library preparation, and RNA sequencing was undertaken in accordance with the procedure described in Cabeza *et al.* (2014).

### Gene expression analysis

For gene expression analysis, the expression level of each gene in each library was calculated by quantifying the number of Illumina reads that mapped to the Mt3.5v3 genome using the Bowtie program, counting only ‘unique hits’. The raw gene expression counts were normalized using the RPKM (reads/Kb transcript length/million total reads) method (Mortazavi *et al.*, 2008). RPKM values were used to compare gene expression within one treatment. Genes showing differential expression between treatment and control were identified using the DESeq method for pair-wise differential expression analysis on values normalized to the effective library size. The DESeq method has proven to be the most reliable way of analysing RNA-seq-based data for differential expression (Dillies *et al.*, 2012). Differentially expressed genes identified by the statistical comparison through the DESeq method were required to have a False Discovery Rate (FDR)-value <0.01. Additionally, either control or treatment tissues were required to have a DESeq value above 20 counts (Supplementary Table S1, at *JXB* online). A validation of the RNA-seq results was performed using qPCR. The qPCR was undertaken according to the Fast SYBR Green Master Mix protocol (Applied Biosystems) on a StepOne™ Real-Time PCR System (Applied Biosystems) following the manufacturer’s recommendations. The primer sequences used are listed in Supplementary Table S3 (at *JXB* online). The measured expression levels correlated between qPCR and RNA-seq with an R^2^ value of 0.86 (Supplementary Table S3.). Over-representation analysis (ORA) of transcripts was carried out using PageMan (Usadel *et al.*, 2006), while the visualization of metabolic pathways was achieved using the MapMan software (Thimm *et al.*, 2004).

### Nodule amino-N compound composition

Around 300mg fresh weight (FW) of nodules was harvested from six plants each that had had a sufficient P supply and six plants that had undergone a 5-day P-depletion period. Nodules were harvested directly into liquid N, extracted, and analysed for amino-N compounds by HPLC, as described in Fischinger *et al.* (2010).

### P concentration in plant DM

Another set of plants in experiment one was harvested for P analyses. The plant material was immediately separated into leaves, stems, roots, and nodules and dried to a constant weight at 65°C. Subsamples of ground plant material were digested in concentrated HNO_3_ at 180°C and the P concentration in the digest was measured colourimetrically using the molybdenum-vanadate method (Scheffer and Pajenkamp, 1952).

### Statistical methods

Statistical analyses other than the analysis of the RNA-seq data were performed using Statistica 10.0 (StatSoft, Inc. Tulsa, USA). When significant differences were detected by ANOVA, the data were tested using Dunnett’s test (*P* < 0.05). For comparison between treatments, the *t*-test was applied (*P* < 0.05).

## Results

### Plant growth and nodule number

**Fig. 2.**
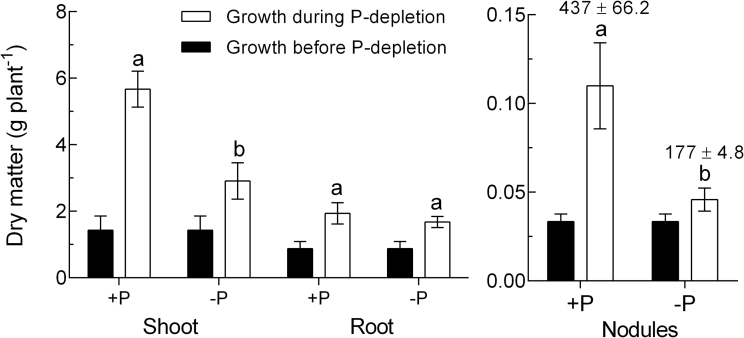
DM of *M. truncatula* plants at the end of the P-depletion experiment. Sufficient P supply (+P) and three weeks of P depletion (–P). Data are given as the mean of five replicates ±SE. Lower case letters indicate a significant difference between treatments (*t*-test, *P*<0.05). Nodule numbers are given above the bars ± SE.

Shoot and root DM in the P-depletion treatment was significantly reduced at the end of the experiment when compared to that of the sufficient-P treatment (Fig. 2). Shoots and nodules were affected much more than the roots. With 10.1g DM plant^–1^ (shoot, roots, and nodules), the plants in the sufficient-P treatment had reached considerable growth after 11 weeks. This growth was reduced to 7g DM plant^–1^ (31% reduction) through the three weeks of P depletion at the end of the growth cycle (see Fig. 1). When considering the increase in DM (shoot, root, and nodules) during the three-week period of P depletion, the incremental biomass accumulation in the P-depletion treatment was reduced by 40% when compared to the control [7.7 vs 4.6g DM (shoot, root, and nodules) in three weeks in the control supplied with sufficient-P versus the P-depletion treatment, respectively]. Nodule DM per plant was strongly reduced by P depletion. While the DM of nodules in the control increased more than 4-fold during the experimental period, the increase in nodule DM was weak in the P-depletion treatment, largely as a result of no new nodules being formed.

### P concentration in the plants during the experiment

**Fig. 3.**
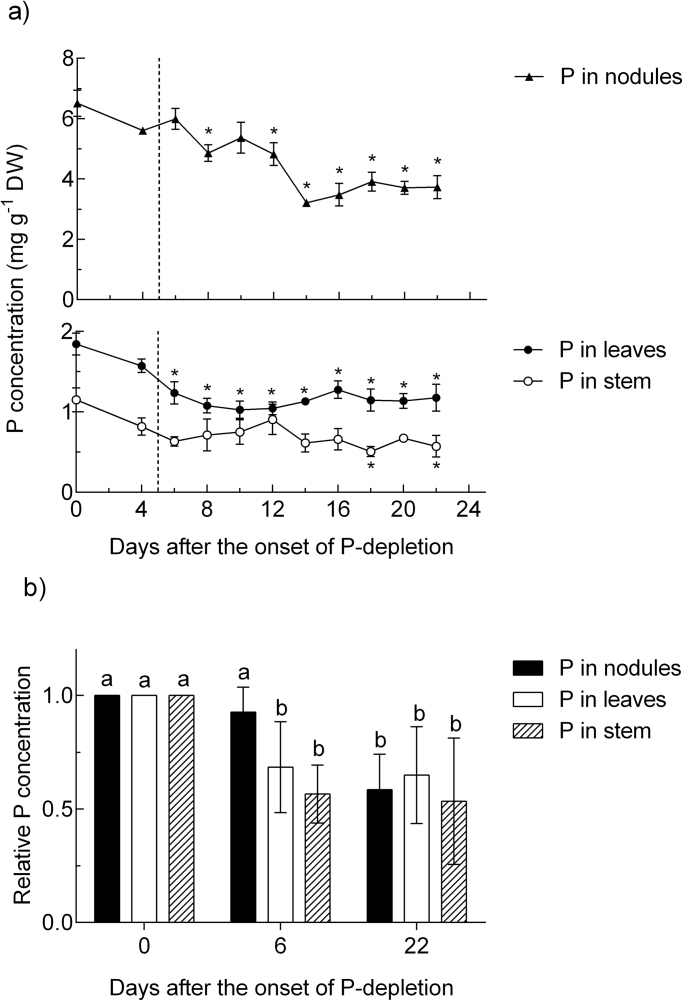
(a) P concentration in different plant organs of *M. truncatula* during whole-plant P depletion. The dotted line indicates the point in time when treatments began to differ significantly in ANA per plant per day. *, indicates a significant difference in comparison with the first day of the experiment (Dunnett test, *P* < 0.05). Data are given as the mean of three replicates ± SE. (b) P depletion in % of the concentration at the beginning of the P-depletion period. Data are from day 6 and 22 after the onset of P depletion.

**Fig. 4.**
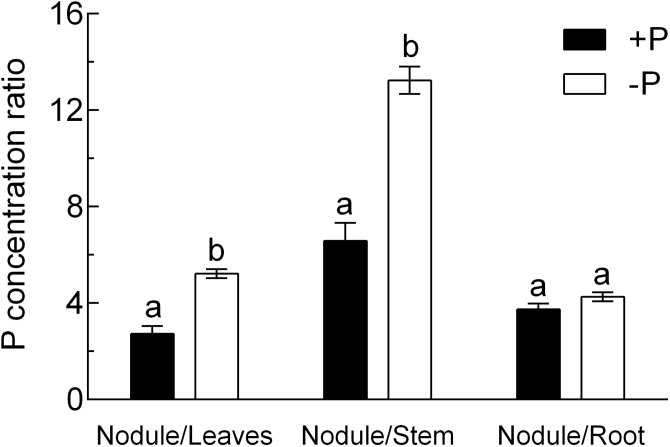
P-concentration ratio of different organs at the end of the P-depletion experiment. Data are given as the mean of five replicates ± SE. Lower case letters indicate a significant difference between treatments (*t*-test, *P*<0.05).

Figure 3a shows the P concentration in leaves, stems, and nodules of the plants during the P-depletion period. In leaves and stems, the decline in P concentration started immediately after the P supply stopped, reaching a lower threshold after 7 days of P depletion. This lower threshold of P concentration was maintained in leaves and stems for the rest of the experimental period. However, the P concentration in stems was not significantly lowered before day 18 of the experiment. The P-depletion pattern in nodules differed markedly in that nodule P concentration remained unaffected during the first 7 days (Fig. 3b), followed by two steps of P dilution from day 7–8 and day 12–15 (Fig. 3a). The P dilution in nodules started later than in leaves, and not before the leaf P concentration had reached the lower threshold. At all points in time in the experiment, nodule P concentration was at least 3-fold that in leaves. This ratio reached a peak at day 7 of the experiment, when the concentration in nodules was about five times that in leaves. The ratio of nodule:leaf and nodule:stem P concentration was significantly higher in the P-depletion treatment at the end of the experiment, while the nodule:root P concentration ratio remained unaffected (Fig. 4).

### Divergence of N_2_ fixation and root/nodule CO_2_ release between the control and treatment during P depletion

**Fig. 5.**
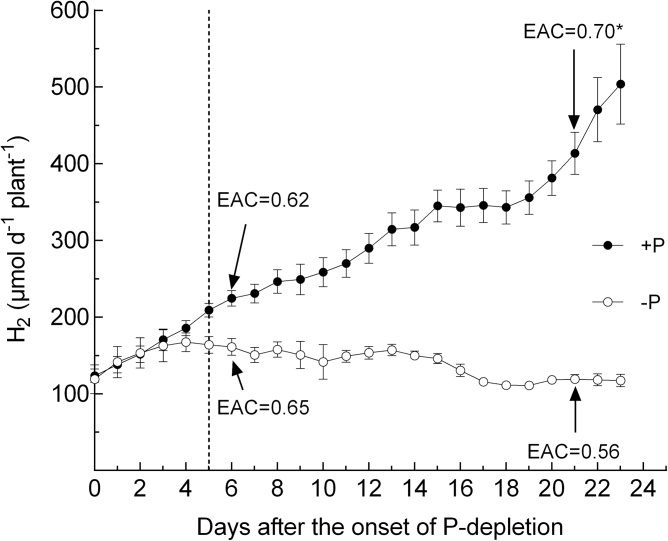
ANA of *M. truncatula* grown with sufficient P supply (closed circles) and during P depletion (open circles). The dotted line indicates the point in time when treatments began to differ significantly in ANA (t-test, *P* < 0.05). * indicates a significant difference in EAC at day 21 (t-test, *P* < 0.05). Data are given as the mean of five replicates ± SE.

**Fig. 6.**
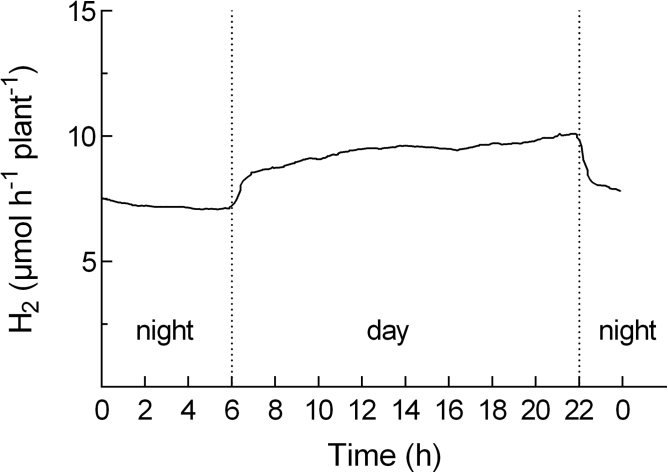
Typical time-course of nodule H_2_ evolution over a 24-h period. Data are the mean of five replicates. The figure shows day 3 of the P-depletion period.

Figure 5 shows the total amount of nodule H_2_ evolution per plant per day (24h) (ANA) over the 23-day P-depletion period in a comparison of the P-depletion treatment and the control. Figure 6 shows a typical time course of nodule H_2_ evolution over a 24-h period (day 3 of the P-depletion period). Data in Fig. 5 are the integral of the time-course of H_2_ release over 24h.

**Fig. 7.**
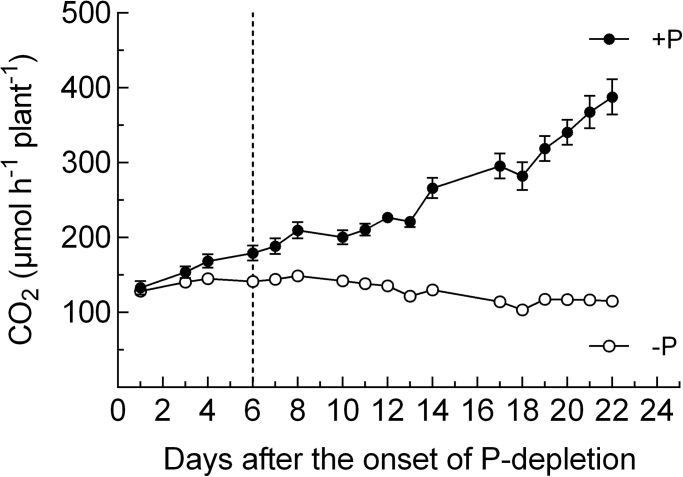
CO_2_ release of roots and nodules of *M. truncatula* grown with sufficient P supply (closed circles) and during P depletion (open circles). The dotted line indicates the point in time when treatments began to differ significantly (*t*-test, *P* < 0.05). Data are given as the mean of five replicates ± SE. Data were taken at 11 am.

On day 5 of the experiment, H_2_ evolution per day (Fig. 5) differed significantly between the treatment and the control. The relative efficiency of nitrogenase (EAC) did not differ between the treatments at that point in time. Subsequently, N_2_ fixation per plant in the P-depletion treatment was maintained at a constant level until day 15 of the experimental period, with a subsequent slight decline during days 16 and 17, and a more or less constant level until the end of the experiment. In contrast, N_2_ fixation in the sufficient-P treatment increased steadily throughout the whole experiment, resulting in approximately a 4-fold increase overall. N_2_ fixation in the sufficient-P treatment showed a high relative efficiency at day 21. At day 21, the relative efficiency (EAC) differed significantly between the treatments and was also significantly lower in the P-depletion treatment when compared to the EAC at day 7 of the experiment. The time course of root/nodule CO_2_ evolution (Fig. 7) confirms the timing of the divergence of both treatments, with a more or less constant level of activity in the P-depletion treatment during the experiment and around a 4-fold increase in the control.

### ANA is not affected during petiole feeding with sucrose

**Fig. 8.**
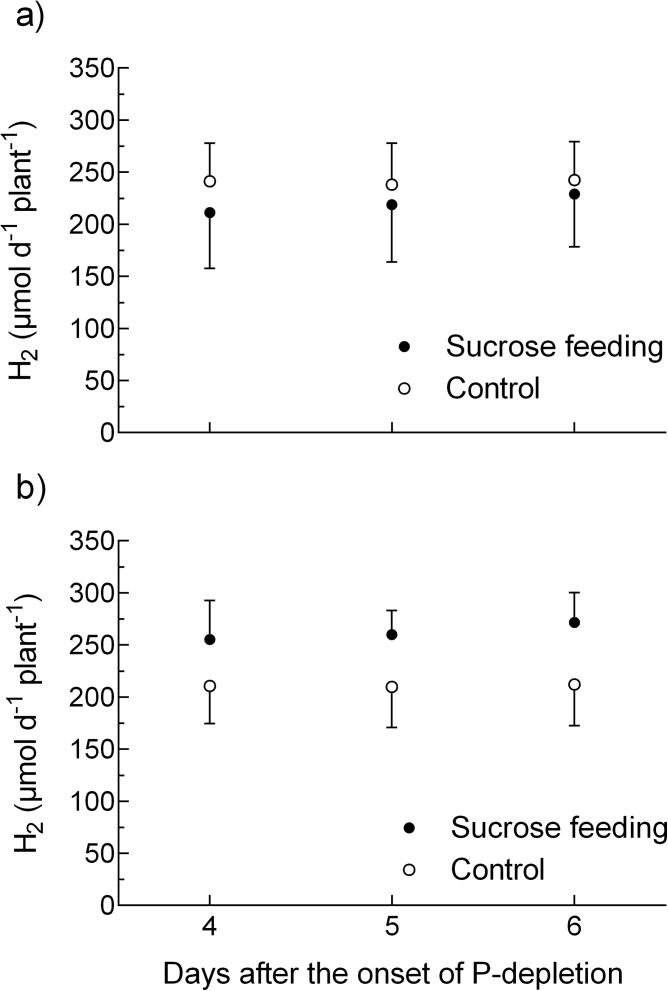
Apparent nitrogenase activity (ANA) of *M. truncatula* plants artificially fed with sucrose: (a) continuous P supply (+P); (b) P depletion (–P). Data are given as the mean of three replicates ± SE.

In a second experiment, a subset of three replicates of plants given an adequate P supply and plants depleted for 4 days were supplied with additional sucrose through an artificial phloem feeding system (petiole feeding; see the material and methods section). The mean additional sucrose uptake was 4.4 mmol C plant^–1^ day^–1^, slightly exceeding the average amount of C respired by roots and nodules at that point in time of the experiment (4.1 mmol C plant^–1^ day^–1^). Figure 8 shows the N_2_ fixation of the four treatments during the period of additional sucrose feeding (days 4–6). Petiole feeding of sucrose did not increase ANA either in plants grown during P depletion or in plants grown with a sufficient P supply.

### Comparative transcriptome profiling (RNA-seq) in treated plants with comparable P concentration but diverging N_2_ fixation

**Fig. 9.**
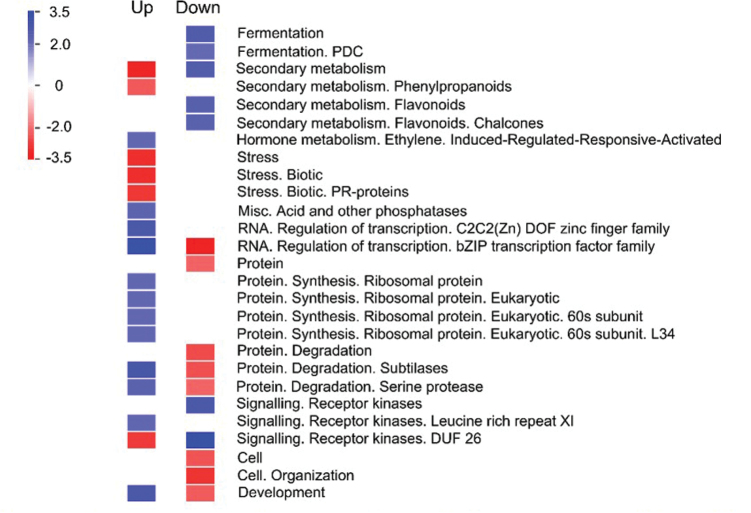
Over-representation analysis (ORA) of genes differentially expressed in nodules of *M. truncatula* at day 5 of whole-plant P depletion. The analysis of representation of differentially expressed genes within functional categories was performed using the PageMan tool of the MapMan software (Thimm *et al.*, 2004) Red, under-represented functional categories; blue, over-represented functional categories. Genes were considered expressed and differentially regulated when they complied with the following criteria: ≥20 ‘unique hit’ counts (DESeq) in treatment and/or control, FDR < 0.01. Data were taken from three biological replicates. The figure is taken from the MapMan software.

In a third experiment, the transcriptome of nodule tissue on day 5 of the whole-plant P-depletion was compared to nodules from fully nourished plants. Nodules were also analysed for free amino acids/amides and sucrose. The RNA-seq yielded 7–17 Mio reads per replicate. About 26 000–28 000 genes of all genes annotated in Mt3.5v3 (47529 genes) were detected. The sequencing therefore reached the necessary depth for this experiment. Only ‘unique hit’ reads were taken as raw counts. Genes with fewer than 20 DESeq normalized counts in control and treatment tissue were considered silent. According to these criteria, 14 617 and 14 330 genes were significantly expressed in control and P-depletion treatment, respectively (Table 1). This corresponds to 30.7 and 30.1% of all genes annotated in the gene model Mt3.5v3 in the control and the P-depletion treatment, respectively. When the DESeq normalized counts were compared between the control and the treatment, a total of 1140 genes were differentially expressed applying an FDR-threshold of 0.01 (n = 3) (Supplementary Table S1). Of these differentially expressed genes, about three quarters were downregulated in the P-depletion treatment (75.4%). An over-representation analysis (ORA) (Fig. 9) shows that various metabolic processes are affected (e.g. genes associated with fermentation processes are over-represented among the downregulated genes, while upregulated genes for stress acclimation are under-represented). The fact that acid and other phosphatases are over-represented among upregulated genes indicates intense P_i_ scavenging and P_i_-remobilization activities due to P limitation of the system. A metabolic overview from a MapMan analysis (Supplementary Figure S1, at *JXB* online) illustrates that the majority of genes involved in primary metabolism were downregulated. Furthermore, a comparatively strong impact on secondary metabolism, in particular on flavonoid and phenylpropanoid metabolism, becomes evident. Supplementary Figure S2 (at *JXB* online) illustrates the number of affected genes involved in regulatory mechanisms at a point in time of P depletion when total P in the nodule tissue itself was unaffected. A high number of transcription factor genes and other genes involved in transcriptional regulation are affected. Furthermore, genes involved in hormone signalling, in particular those in connection with ethylene, and genes for protein turnover and receptor kinases, are differentially expressed. Overall, despite the fact that actual P depletion had not yet commenced in the nodules, the whole-plant P depletion had already made a considerable impact on the molecular network of the nodules, in particular at the regulatory level.

**Table 1.** Gene expression in nodules of *M. truncatula* in undisturbed nodules and in nodules after 5 days of whole-plant P depletion^a^

	Total genes expressed	Upregulated (–P)	Downregulated (–P)
Control	14 617	280	860
–P	14 330		

^a^ Genes were considered expressed when they complied with the following criteria: ≥20 ‘unique hit’ counts (DESeq) in treatment and/or control and differentially regulated when the False Discovery Rate (FDR) was <0.01. Data were taken from three biological replicates.

### Differential expression of genes that typically indicate P deficiency

Although the total P concentration of the nodules analysed had not declined in the P-depletion treatment when the analyses were conducted, various genes known to react to P deficiency were differentially regulated, while the expression of genes related to N metabolism remained largely unchanged. A group of genes known to react to P deficiency (Li *et al.*, 2002) that mobilizes P from organically bound forms was strongly induced. Two genes for a pyridoxal phosphate phosphatase (AC235757_50.1, AC235757_9.1), a gene for a putative pyridoxal phosphate phosphatase (AC235757_5.1), and a gene for a purple acid phosphatase (Medtr6g013050.1) were upregulated (Supplementary Tables S1 and S2). This indicates that the nodules begin to acclimate to the overall scarcity of P by increasing P turnover. Since only total P was measured, it cannot be ruled out that at that point in time of the depletion process, shifts in the inner nodule relative P allocation to the individual P fractions had already occurred. Additionally, the upregulation of a gene for a PHO1-like protein (Medtr2g077690.1, log_2_ FC +2.12, RPKM control 3.2) indicates P deficiency and the start of phosphorus mobilization from the nodules. The protein is involved in phosphorus loading of the xylem (Hamburger *et al.*, 2002). However, a gene from the ubiquitin-conjugating enzyme family (Medtr2g013650.1) that closely resembles *PHO2* of *Arabidopsis* (AT2G33770.1) was upregulated (log_2_ FC +0.82, RPKM control 18.9). In *Arabidopsis*, the *PHO2* is regulated by a shoot-borne miRNA that is formed in dependence on the shoot P status and mediates the turnover of a central inorganic P transporter (AtPHT1.1) (Lin *et al.*, 2008; Chiou and Lin, 2011). A sequence comparison revealed an 86% identity of the coding sequences of both genes in *Arabidopsis* and *M. truncatula*. Out of nine genes annotated as inorganic phosphate transporters three were expressed in the nodules of *M. truncatula* in our experiments. None of these three genes was differentially regulated. The highest expression was found for Medtr1g074930.1 (Supplementary Table S2). A sequence comparison shows that the gene is closely related to GmPT5, which was shown to be of importance for P import into soybean nodules (Qin *et al.*, 2012). Two genes for unknown proteins which, however, contain a conserved region of the phosphate-induced protein (*phi1*) in tobacco (Sano *et al.* 1999) were strongly downregulated (Medtr5g064340.1, log_2_ FC –1.86, RPKM control 44; Medtr5g064360.1, log_2_ FC –2.85, RPKM control 68). In contrast, genes known to be transcriptionally regulated in response to changes in the N metabolism remained unchanged. For example, two genes for the nodule inception protein, known to be involved in N metabolism (Castaings *et al.*, 2009), were expressed and remained unchanged by the treatment (Supplementary Table S1). In a related paper, the inhibitory effect of nitrate on nodules was connected to strong effects on the expression of genes of NCR peptides, leghaemoglobins, and nicotianamine synthase (Cabeza *et al.*, 2014). The nodules from the P-depleted plants showed none of these reactions and the expression of the above-mentioned genes and gene families remained unchanged (Table S1 and S2).

### Amino-N compounds and sucrose concentration in nodules in treated plants with similar nodule P concentration but diverging N_2_ fixation

The concentration of various amino acids/amides in nodules is shown in Table 2. Amino-N compounds were detected when the concentration was above a detection threshold of 0.05 µmol g^–1^ nodule FW. Ten amino-N compounds were measured in a concentration above that threshold (Table 2). The bulk of this fraction (77–80%) was asparagine. The total concentration of amino-N compounds was significantly lower in nodules of the P-depleted treatment, largely as a result of less asparagine. Asparagine is the main form of N-export from nodules of the indeterminate type (Fischinger and Schulze 2010*b*). The second most abundant amino-N compound found in nodules of both treatments was gamma-aminobutyric acid (GABA). The concentration of sucrose in nodules at day 5 of the P-depletion process was significantly reduced by about 25% (23.2 and 17.6 µmol g^–1^ FW nodule in +P and –P plants, respectively) (t-test, n = 6, *P* < 0.05).

**Table 2.** Amino acid composition in the nodules of *M. truncatula* plants grown at sufficient P supply (+P) and under P depletion (–P)

	+P		–P	
	µmol g^–1^ FW	%	µmol g^–1^ FW	%
Aspartic acid	1.2±0.3	4.0	0.7±0.1	4.2
Asparagine	23.3±7.9	80.0	12.2±1.7	77.0
Alanine	0.5±0.1	1.8	0.6±0.1	3.9
γ-Aminobutyric acid	2.8±0.5	9.6	1.3±0.2	8.3
Tyrosine	0.2±0.0	0.6	0.1±0.0	0.7
Methionine	0.1±0.0	0.3	0.1±0.0	0.5
Valine	0.4±0.1	1.5	0.4±0.0	2.4
Phenylalanine	0.2±0.0	0.5	0.1±0.0	0.7
Isoleucine	0.2±0.0	0.8	0.2±0.0	1.1
Leucine	0.2±0.1	0.8	0.2±0.0	1.1
Total	29.1	100	15.9	100

Data are given as mean of four replicates ± SE.

## Discussion

### N_2_ fixation of existing nodules is maintained during P depletion, but the formation of new nodules ceases

P is crucially important for active legume nodules and high N_2_-fixation rates. P deficiency has a negative impact on N_2_ fixation of legumes and various authors have shown that legume growth under P shortage is linked to reduced N_2_ fixation (Leidi and Rodríguez-Navarro, 2000; Tang *et al.*, 2001; Høgh-Jensen *et al.*, 2002; Graham *et al.*, 2003). This study shows that the plants maintain high N_2_-fixation of existing nodules when leaf P concentration is greatly reduced. At day 5 after the cessation of P supply, nodule P concentration was unchanged while leaf P concentration was significantly reduced. The two treatments differed significantly in N_2_ fixation per plant from day 5 onwards. However, the plants maintain the activity of the existing nodules at a considerable level, while on a whole-plant basis N_2_ fixation was restricted by a virtual cessation of the formation of new nodules during the P-depletion process. The nodule metabolism at day 5 of the depletion experiment consequently represents a key moment at which the whole plant is strongly affected by P deficiency, but processes of acclimation enable the plant to maintain the activity of existing nodules. At that point in time after the cessation of P supply, a strong shift in the transcriptome had occurred, with a significantly altered expression of 1140 genes. A MapMan analysis of altered gene expression reveals numerous effects on regulatory processes involving hormones, transcription factors, receptor kinases, redox and calcium-related effects, and protein modification and turnover (Supplementary Figure S1 and Table S1). During the subsequent time course of this experiment, N_2_ fixation per plant was maintained at a constant level from days 8–15, and was not fully lost during the rest of the experimental period. It is known that legumes under mild P-deficiency display a deeper green colour and increased N concentration (Almeida *et al.*, 2000). Nevertheless, the high P-concentrations required in nodules (Jakobsen, 1985), in conjunction with intensive nodule respiration (Hunt and Layzell, 1993) and the pivotal importance of P for photosynthesis (Fredeen *et al.*, 1990), make it appear feasible that the initial limitation of legume growth at low P-supply is a restriction of N_2_ fixation. In a detailed study of Israel (1993) about the sequence of events that occur during the onset of P deficiency, decreased N_2_-fixation and lower N-concentration in the plants were among the early reactions. Moreover, in a field-scale study performed on a strongly P-depleted soil, legume growth was primarily restricted by lower N_2_-fixation and P fertilization strongly increased N_2_ fixation. This might be explained by the fact that the P availability in that study was extremely low from early on in the plant development (Römer and Lehne, 2004). Usually plants might suffer a P deficiency that develops more slowly. Our experiments were intended to mimic such conditions and the data show that plant growth was not limited by N_2_ fixation at any point in the experiment. By contrast, the plants appear to follow a strategy of adapting the existing nodules by various measures, to store N, and to maintain viable leaf tissue until, for example, root-initiated active soil-P mobilization processes take effect and support further growth.

After day 8 of P depletion, both the stem and leaves reached a lower threshold of 0.6 and 1.1mg P g DM^–1^, respectively. This threshold concentration remained unchanged during the rest of the experiment. It appears that this level of P concentration is necessary for *M. truncatula* to preserve viable tissue, at least for a certain period of time. In fact, a significant amount of necrotic leaves was not observed before 15 days of P depletion. After day 8, when the lower threshold of P concentration had been reached in shoots, some P remobilization (25%) from nodules began. Although the P concentration of nodules was about three times that of leaves, as a proportion of total plant P it was negligible (about 0.5% of P in shoots). Thus the fact that no further dilution of leaf P occurred after day 8 and the remobilized total amount of P from nodules was very low indicates that during that period of the experiment, plant growth had virtually ceased or was at least very slow in the P-depletion treatment. Overall these data enhance our understanding of the allocation of scarce P in legumes with the observation that although P concentration is much higher in nodules, any dilution in or remobilization from this organ would not commence before the shoot concentration had reached the lower threshold apparently necessary to maintain viable tissue.

### Assimilate supply, C turnover and ATP formation do not limit N_2_ fixation

**Fig. 10.**
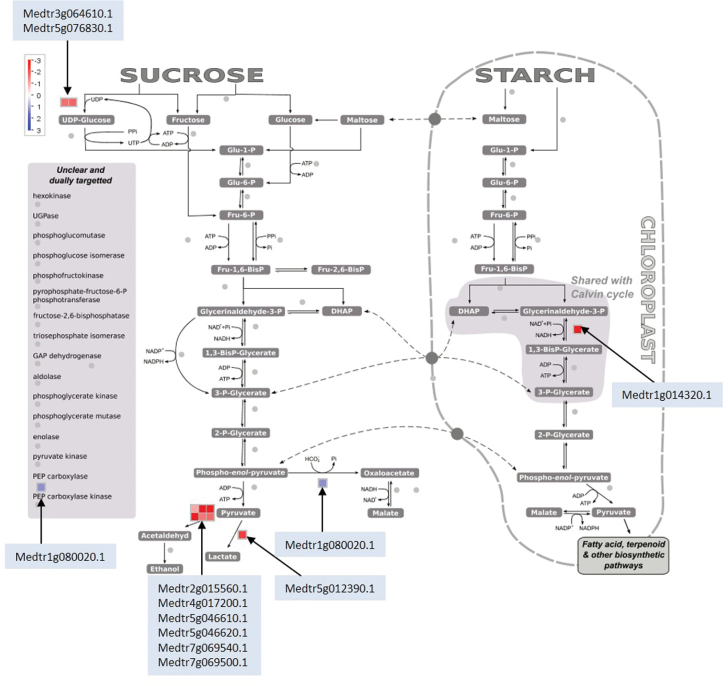
MapMan analyses of gene expression in the glycolytic pathway. Blue bins represent upregulation and red bins represent downregulation. The figure shows that fermentation processes were strongly downregulated and nodule CO_2_ fixation through PEP carboxylase was upregulated. This represents a potentially P-saving alternative glycolytic flux. A further potential P-saving regulation is the lower expression of an NADP-dependent glyceraldehyde-3-phosphate dehydrogenase. Differentially expressed genes and the DESeq normalized counts are listed in Supplementary Table S1. Genes were considered expressed and differentially regulated when they complied with the following criteria: ≥ 20 ‘unique hit’ counts (DESeq) in treatment and/or control, FDR < 0.01. Data were taken from three biological replicates. Gene expression is visualized using the MapMan software (Thimm *et al.*, 2004).

At the time of the experiment when N_2_ fixation was affected (day 5), nodule sucrose concentration had decreased by about 25%. Nevertheless, the maintained level (17.6 µmol g^–1^ FW nodule) still exceeds literature data for sucrose concentration in undisturbed nodules [e.g. ~11 µmol g^–1^ FW nodule for *M. truncatula* (Sulieman *et al.*, 2013), 7–18 µmol g^–1^ FW nodule for pea (Fischinger and Schulze, 2010*b*), and ~8 µmol g^–1^ FW nodule for soybean (Streeter, 1987)]. In addition, artificial sucrose feeding into the phloem when the leaves were already strongly P depleted had no effect. Although significant amounts of sucrose were taken up, this resulted neither in a delay of the divergence of the treatment in N_2_ fixation per plant nor in increased specific N_2_-fixation activity in the treatment and control. It appears that assimilate availability can buffer interrupted or disturbed CO_2_ assimilation for long periods. As an example, throughout the entire experimental period the plants were able to maintain significant and constant N_2_ fixation during each day’s 8-hour dark period in the growth chamber. This was also the case in the P-depletion treatment during the whole depletion period. The ability of legumes to adapt and buffer assimilate supply to nodules is supported by the observation that the specific nodule respiration driving nitrogenase activity can vary widely (Schulze, 2004). Where conditions are undisturbed, assimilate consumption is much higher than what has been calculated assuming the most efficient assimilate for ATP and reductant conversion. Moreover, the C efficiency of N_2_ fixation can be much greater in the same plants during periods of greater inner plant competition for assimilate (e.g. during pod filling) (Adgo and Schulze, 2002). The transcriptome profiling data overall support the observation that assimilate supply and respiration is not limiting at day 5 of the P-depletion process. A MapMan analysis (Fig. 10) reveals that, with the exception of a gene for NADP-dependent glyceraldehyde-3-phosphate dehydrogenase (Medtr1g014320.1), glycolysis and the citric acid cycle (not shown) in nodules remained unaffected. Furthermore, transcript abundance of genes for the mitochondrial electron transport chain remained constant, with the exception of one gene for cytochrome c (Medtr5g008460.1) that was slightly downregulated (log_2_ FC of –0.67, RPKM control 281). In addition, after 5 days of whole-plant P depletion, a gene for the mitochondrial chaperone BCS1 (Medtr3g092060.1, log_2_ FC of –0.85, RPKM control 26) was downregulated. The chaperone is involved in the assembly of complex III of the mitochondrial electron transport chain (Heazlewood *et al.*, 2004). Two genes for ATP synthase subunit beta (Medtr1g075960.1, log_2_ FC of –2.81, RPKM control 0.68; Medtr8g037100.1, log_2_ FC of –1.19, RPKM control 57) were downregulated. Both genes are annotated as chloroplastic in the gene model Mt3.5v3. Other genes for ATP synthase, in particular those found in the mitochondrial inner membrane, remained unaffected (Supplementary Table S1 and S2). Sucrose synthase (SS) is a key enzyme for nodule functioning (Baier *et al.*, 2007). A downregulation of two SS genes (Medtr3g064610.1 and Medtr5g076830.1) occurred after 5 days of P depletion. With RPKM values of 4.6 and 9.1, respectively, these genes were comparatively low in transcript abundance, yet were the strongest expressed genes for SS in nodules (Table S2). According to this study’s threshold (≥20 DESeq in control and/or treatment tissue) an additional seven genes for SS were expressed in nodules, but not differentially regulated, and a further eight genes remained silent. However, several genes for invertases were expressed and remained unchanged by the treatment, in particular a neutral invertase-like protein (Medtr1g096140.1), with a comparatively high level of expression (133 RPKM). Thus our data support a possible role played by invertases in nodule sucrose metabolism (Tesfaye *et al.*, 2007; Moreau *et al.*, 2011). No genes for sucrose transporters were found to be affected by the emerging P deficiency (Supplementary Table S1 and S2).

### Differentially expressed genes indicate a downregulation of nodulation

Although this study was aimed at the reactions of nodules to P depletion, some differentially regulated genes involved in nodulation are worth mentioning. Eight genes for chalcone synthase were strongly downregulated (Supplementary Table S1). Chalcone synthase is a key enzyme in flavonoid biosynthesis (Dao *et al.*, 2011). Silencing of the gene results in strongly reduced nodulation (Wasson *et al.*, 2006; Abdel-Lateif *et al.*, 2013). Overall, several genes of the phenylpropanoid pathway were downregulated (Supplementary Table S1). A further indication that nodule formation was reduced is the upregulation of the receptor protein kinase *CLV1* (Medtr4g070970.1) (Supplementary Table S1). A mutation in that gene results in the supernodulating Mt_SUNN_ phenotype (Schnabel *et al.*, 2005). P deficiency is known to induce senescence in legume nodules (Sulieman *et al.*, 2013). Ethylene is possibly involved in nodulation and nodule senescence (Puppo *et al.*, 2005; Van de Velde *et al.*, 2006; Vernie *et al.*, 2008). There are strong indications in this study’s data set that ethylene biosynthesis is repressed. Five genes for ACC oxidases and one gene for ACC synthase were downregulated (Supplementary Table S1). This might constitute a further measure by the plants to uphold the activity of existing nodules, counteracting the effects associated with senescing P-depleted tissue.

### Expression of genes involved in nodule CO_2_ fixation and malate formation is enhanced

Figure 9 shows that the C flow is already strongly redirected from fermentation processes to increased formation of malate after 5 days of P depletion. Several genes involved in fermentation were strongly downregulated from a high level of expression. This applies to six genes for pyruvate decarboxylase (average log_2_ FC of –2.44, RPKM control 186), two for alcohol dehydrogenase (average log_2_ FC of –2.98, RPKM control 65), two for quinohaemoprotein ethanol dehydrogenase (average log_2_ FC of –1.95, RPKM control 56), and one for L-lactate dehydrogenase (log_2_ FC of –2.28, RPKM control 281). At the same time, two genes for phosphoenolpyruvate carboxylase (PEPC) (Medtr1g094010.1, Medtr2g076670.1) were upregulated with a log_2_ FC of +1.26 and +0.73, respectively. The transcriptome data reveal that in addition to the known fact of increased CO_2_ dark fixation of nodules under P stress, a concerted downregulation of fermentation processes channels further C into malate formation. One gene for PEPC (Medtr2g076670.1, RPKM control 421) is much more highly expressed in the control than any other member of the gene family (Supplementary Table S2). The gene is therefore a candidate for the nodule-enhanced form of PEPC in *M. truncatula* (Pathirana *et al.*, 1992). PEPC is a key enzyme for malate formation which is the principal C and energy source for bacteroids and the C skeleton for asparagine, the major form in which N is allocated to shoots (Fischinger and Schulze, 2010*b*). The two additional enzymes involved in nodule CO_2_ fixation and malate formation remained largely unaffected. Five genes for carbonic anhydrase were found to be expressed in the nodules, among which a gene for a bifunctional monodehydroascorbate reductase/carbonic anhydrase nectarin-3 (Medtr7g090950.1) was downregulated (log_2_ FC –0.87, RPKM control 14) and one gene (Medtr3g077910.3) upregulated (log_2_ FC +0.90, RPKM control 1.7) (Supplementary Tables S1 and S2). Five genes for malate dehydrogenase were expressed. The expression remained unaffected by the P depletion. Among the MDH-genes, the transcript of Medtr1g043040.1 was by far the most abundant (RPKM control 255) (Supplementary Tables S1 and S2). Malate formation, and in particular nodule CO_2_ fixation through PEPC, is of vital importance for nodules. Silencing of PEPC in *M. sativa* nodules resulted in strongly decreased nodule activity (Schulze *et al.*, 1998). Under P stress, increased nodule malate production has been reported (Le Roux *et al.*, 2006). The process represents a C- and energy-saving pathway for the nodules (Fischinger and Schulze, 2010*b*) and furthermore can circumvent the P-dependent pyruvate kinase activity (Vance *et al.*, 2003). Pyruvate kinase can be severely affected by P stress (Plaxton and Tran, 2011). In this study’s experiments, the transcript abundance of the six expressed genes for pyruvate kinase was not affected. Other potential alternative glycolytic reactions (Vance *et al.*, 2003) circumventing reaction steps that need P were not upregulated in the nodules, indicating that these processes (e.g. the PP_i_-dependent phosphofructokinase) are not induced before the actual level of P in the tissue declines.

### Proteinase inhibitors are downregulated

Among the ten most downregulated genes were three proteinase inhibitors that contain the Kunitz domain (Supplementary Table S1). The two most downregulated genes were a gene annotated as 21kDa seed protein (log_2_ FC of –8.87, RPKM control 252) and a pathogen-inducible trypsin-inhibitor-like protein (log_2_ FC of –6.99, RPKM control 56). Both proteins contain the Kunitz domain, which is a domain that defines a proteinase inhibitor protein family (Rawlings *et al.*, 2004). Furthermore, two genes for miraculin were downregulated with a high fold-change and considerable initial transcript abundance (Medtr6g078280.1 log_2_ FC of –6.45, RPKM control 357; Medtr6g078290.1. log_2_ FC of –3.52, RPKM control 1635). Miraculin itself is a glycoprotein, possesses trypsin inhibitory activity and is involved in the plant’s response to pathogens (Hiraoka *et al.*, 2009; Selvakumar *et al.*, 2012). On the human tongue, the protein pH-dependently activates the activity of a sweet taste receptor (Koizumi *et al.*, 2011). The role of these proteinase inhibitors in nodules is not fully understood. They are involved in defensive measures of the higher plants in that they inhibit proteases exuded by an invading pathogen (Hörger and van der Hoorn, 2013). In the case of nodules they might be necessary for keeping the bacteria inside the nodule and the infected cells (Lievens *et al.*, 2004). To the authors’ knowledge, the fact that this group of genes for proteinase inhibitors shows a strong decrease in transcript abundance in nodules under P deficiency has not yet been described. However, the functional implications remain unclear. Genes for that family of proteinase inhibitors were inversely regulated in a related study (Cabeza *et al.*, 2014), under a treatment that is known to decrease N_2_ fixation (nodule exposure to nitrate).

In conclusion, during whole-plant P depletion, nodules maintain constant total P concentrations until the P dilution in leaves has reached a lower threshold that is probably indispensable for the tissue to remain viable. During that period, strong shifts of the nodule metabolism occur which acclimate the nodules to low P availability before the tissue itself is depleted. Among these mechanisms, increased CO_2_ fixation as well as reduced activity of fermentation pathways and upregulation of phosphatases that will mobilize P from organic structures and thus would contribute to a higher turnover of the scarce P are important. These measures enable the plant to uphold the high N_2_-fixation rates of existing nodules well into the P-depletion process. In fact, at no point in time of the inner plant P-depletion process is plant growth limited by reduced N_2_ fixation. This strategy necessitates maintaining a threshold of P concentration in the leaves that enables them to provide a sufficient assimilate supply of the nodules. The interdependent leaf (N from nodules) and nodule (C from leaves) tissue and their source–sink relations undergo stepwise changes during P depletion maintaining a functioning system as long as possible. The main conclusion from our experiments is that efforts for improving legume performance under P deficiency should target processes that allow and promote maintenance of the plants’ leaf CO_2_ fixation and nodule N_2_ fixation pathways further into the P-depletion process. Plant material with a lower P concentration threshold of leaves (higher internal P use efficiency) would in particular be promising, not only for plant growth but also for N_2_ fixation under conditions of limiting P.

## Supplementary material

Supplementary data can be found at *JXB* online.

Supplementary Table S1. Transcripts differentially expressed between untreated nodules and nodules after 5 days of P depletion.

Supplementary Table S2. RPKM values for all genes annotated in Mt3.5v3 in untreated nodules and nodules after 5 days P depletion.

Supplementary Table S3. qPCR-validation of the RNA-seq data.

Supplementary Figure S1. Metabolic overview showing differences in gene expression between P-deficient and control nodules of *M. truncatula.*

Supplementary Figure S2. Regulation overview showing differences in gene expression between P-deficient and control nodules of *M. truncatula.*

## Funding

This work was supported by a grant of the German National Science Foundation (DFG SCHU 1602/7-1). Ricardo A. Cabeza was supported by a postdoctoral fellowship from the Chilean Government (Becas Chile programme).

## Supplementary Material

Supplementary Data
