# Supplementary material for: RNA-seq transcriptome profiling reveals that Medicago truncatula nodules acclimate N2 fixation before emerging P deficiency reaches the nodules
Source: J Exp Bot. 2014 Aug 23;65(20):6035–48. doi: 10.1093/jxb/eru341 (PMC4203135; doi:10.1093/jxb/eru341)
Supplement: Supplementary Data [file supp_65_20_6035__index.html]

RNA-seq transcriptome profiling reveals that Medicago truncatula nodules acclimate N2 fixation before emerging P deficiency reaches the nodules — Supplementary Data 

# RNA-seq transcriptome profiling reveals that *Medicago truncatula* nodules acclimate N2 fixation before emerging P deficiency reaches the nodules

## Supplementary Data

Data files

**Files in this Data Supplement:**

- Supplementary Data - Supplementary Data
- Supplementary Data - Supplementary Data
