# Supplementary material for: RNA-seq transcriptome profiling reveals that Medicago truncatula nodules acclimate N2 fixation before emerging P deficiency reaches the nodules
Source: J Exp Bot. 2014 Aug 23;65(20):6035–48. doi: 10.1093/jxb/eru341 (PMC4203135; doi:10.1093/jxb/eru341)
Supplement: Supplementary Data [file supp_eru341_jexbot123885_file002.pdf]

RNA-seq transcriptome profiling reveals that *Medicago truncatula* nodules adapt N_2 fixation before emerging P deficiency reaches the nodules

Ricardo A. Cabeza, Rebecca Liese, Annika Lingner, Ilsabe von Stieglitz, Janice Neumann, Gabriela Salinas-Riester, Claudia Pommerenke , Klaus Dittert and Joachim Schulze

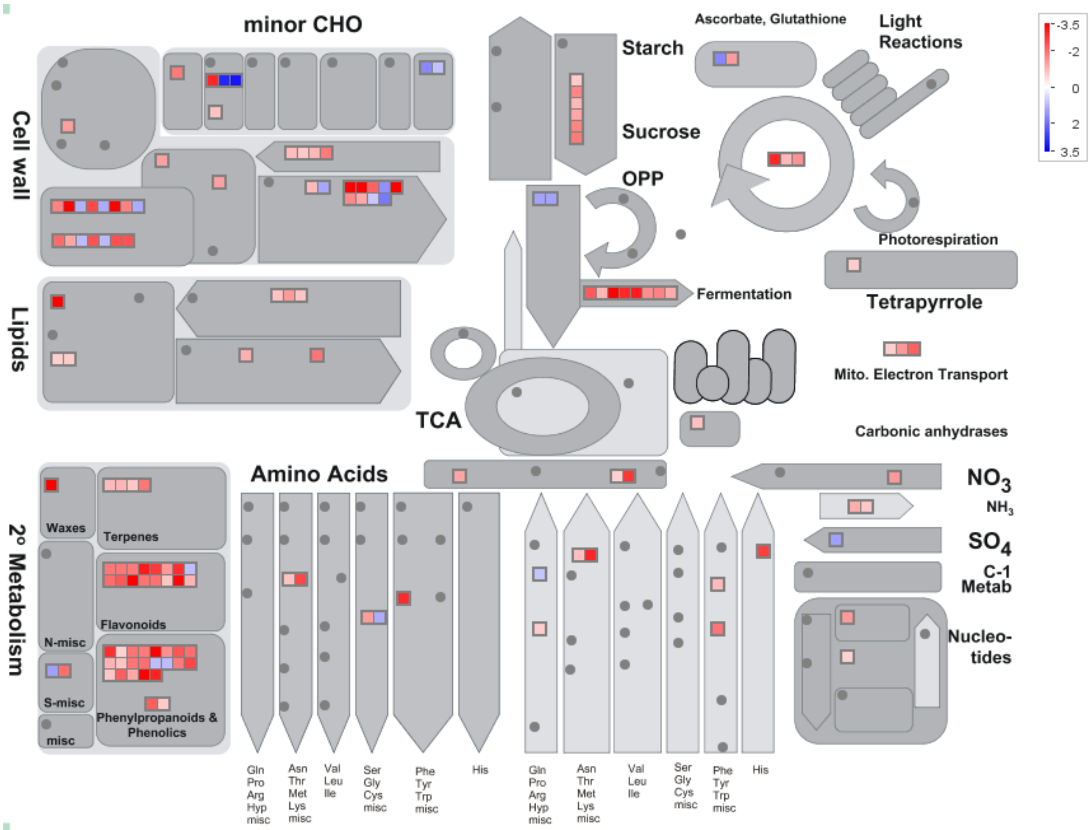

Supplemental material Figure S1. Metabolic overview showing differences in gene expression between nodules of P-deficient *M. truncatula* plants and control nodules.

In the colour scale, blue represents up regulation and red represents down regulation. Differentially expressed genes and the DEseq normalized counts are listed in Supporting Information TableS1. Genes were considered expressed and differentially regulated when they complied with the following criteria: ≥ 20 ´unique hit` counts (DEseq) in treatment and/or control, FDR < 0.01. Data were taken from three biological replicates. The figure is taken from the MapMan software (Thimm *et al*., 2004).

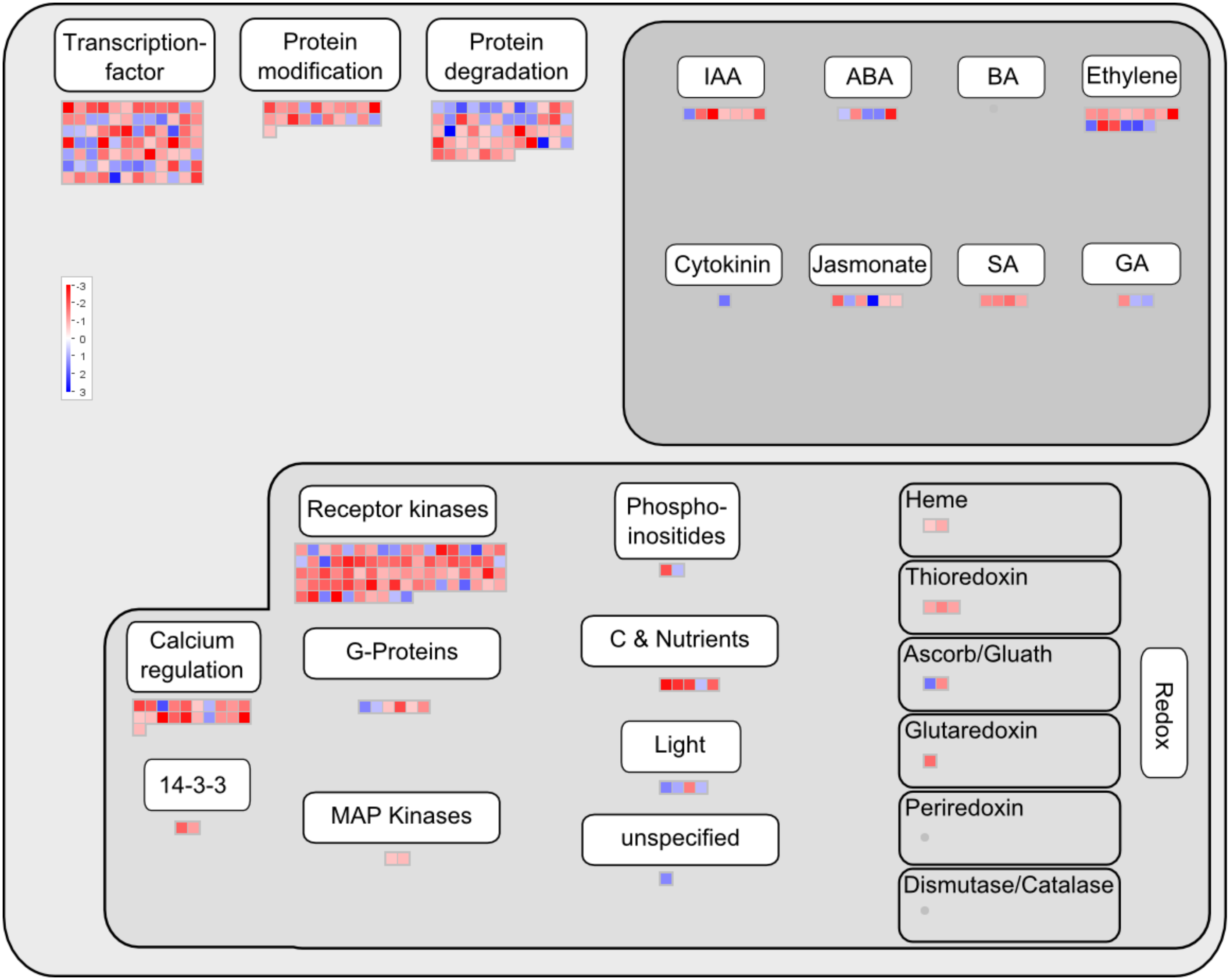

Supplemental material Figure S2. Regulation overview showing differences in gene expression between P-deficient and control nodules of *M. truncatula.*

In the colour scale, blue represents up regulation and red represents down regulation. The figure illustrates that the majority of the differentially regulated genes showed lower transcript abundance. Metabolic regulations occur in particular through differentially regulated hormone related genes, transcription factors, receptor kinases, calcium-dependent processes and genes involved in protein modification and degradation. Gene expression is visualized through the MapMan software (Thimm *et al*., 2004).
